# Chromosome number evolves at equal rates in holocentric and monocentric clades

**DOI:** 10.1371/journal.pgen.1009076

**Published:** 2020-10-13

**Authors:** Sarah N. Ruckman, Michelle M. Jonika, Claudio Casola, Heath Blackmon

**Affiliations:** 1 Department of Biology, Texas A&M University, Texas, United States of America; 2 Ecology and Evolutionary Biology Interdisciplinary Program, Texas A&M University, Texas, United States of America; 3 Genetics Interdisciplinary Program, Texas A&M University, Texas, United States of America; 4 Department of Ecology and Conservation Biology, Texas A&M, Texas, United States of America; Stowers Institute for Medical Research, UNITED STATES

## Abstract

Despite the fundamental role of centromeres two different types are observed across plants and animals. Monocentric chromosomes possess a single region that function as the centromere while in holocentric chromosomes centromere activity is spread across the entire chromosome. Proper segregation may fail in species with monocentric chromosomes after a fusion or fission, which may lead to chromosomes with no centromere or multiple centromeres. In contrast, species with holocentric chromosomes should still be able to safely segregate chromosomes after fusion or fission. This along with the observation of high chromosome number in some holocentric clades has led to the hypothesis that holocentricity leads to higher rates of chromosome number evolution. To test for differences in rates of chromosome number evolution between these systems, we analyzed data from 4,393 species of insects in a phylogenetic framework. We found that insect orders exhibit striking differences in rates of fissions, fusions, and polyploidy. However, across all insects we found no evidence that holocentric clades have higher rates of fissions, fusions, or polyploidy than monocentric clades. Our results suggest that holocentricity alone does not lead to higher rates of chromosome number changes. Instead, we suggest that other co-evolving traits must explain striking differences between clades.

## Author summary

One of the most basic features of the genome is its division into chromosomes. When we look across clades, we find a striking pattern where almost all species in some groups exhibit the same number of chromosomes, while in other groups, there is great variation in chromosome number even between closely related species. One possible explanation for these differences is the type of centromeres that a clade possesses. Monocentric chromosomes must have a single region that functions as the centromere, while in holocentric chromosomes, centromere activity is present across the entire chromosome. For this reason, it has been hypothesized that holocentric species should tolerate structural changes in chromosomes (fusions, fission, etc.) better than monocentric species. To test this hypothesis, we estimated the rate of fusion, fission, and polyploidy using data from 4,393 species of insects spanning 22 orders. We found that insect orders exhibit striking differences in rates of chromosome evolution. However, across all insects, we found no evidence that holocentric orders have higher rates of fissions, fusions, or polyploidy than monocentric orders. Our results suggest that holocentricity alone does not lead to higher rates of chromosome number changes.

## Introduction

Chromosome number stability is generally expected among lineages as shifts in chromosome number can lead to a decrease in fitness [1–3]. This stability in chromosome number is thought to be driven by the underdominance of chromosomal rearrangements [4]. However, this expected stability is challenged by some clades that exhibit striking variation in chromosome number as well as the interdigitation of fast and slow evolving lineages within clades [5–7]. Understanding the reasons that clades vary in rates of chromosome number evolution is key to understanding how genome structure evolves. Furthermore, changes in chromosome number and less drastic changes like inversions can play a key role in divergence, adaptation, and speciation [2, 8–10]. In light of the potential impacts of chromosomal change, identifying traits associated with increased rates of chromosomal rearrangements is a key step in understanding patterns of extant diversity.

Within clades, karyotypes are often reshaped through fusions and fissions [11]. We use these terms (fusion and fission) for simplicity to describe increases or decreases of chromosome number by one. However, in reality, fusions decreasing chromosome number capture two different processes at the molecular level. First, translocations followed by the possible loss of a small fragment of one chromosome can decrease chromosome number (e.g. Robertsonian translocation in monocentric species) [12]. Second, the fusion of telomeres from two chromosomes (in monocentric species this would also require the inactivation of one of the ancestral centromeres), as evidenced by the evolutionary history of human chromosome 2 [13]. In contrast, changes increasing chromosome number can occur through simple fissioning in the centromere region and the gain of new telomeric sequences [12, 14]. Increases in chromosome number can also occur through polyploidy or aneuploidy. In the case of polyploidy, the numbers of copies of the genome will increase by one from fertilization of an unreduced gamete [15]. Likewise, aneuploidy can lead to the duplication of single chromosomes [16].

Because chromosomal rearrangements are thought to often be deleterious or underdominant, they should be more likely to fix in populations with meiotic drive or low effective population size [5, 17, 18]. However, centromeric structure may modulate the fitness effects of fusions and fissions [19, 20]. In species with monocentric chromosomes fusions and fissions can lead to multiple or no centromeres along the length of a chromosome which leads to failed segregation [21, 22]. In contrast, the centromeres in holocentric species are diffuse and spindle fibers attach along the entire length of the chromosome. In these species, fusions and fissions do not appear to disrupt proper segregation [23–26]. Therefore, holocentricity has the potential to reduce or eliminate selective pressure against chromosomal rearrangements. This should lead to higher rates of chromosome number evolution in clades with holocentric chromosomes relative to clades with monocentric chromosomes. However, results from studies of individual holocentric clades have been mixed, with some clades showing great variation [27, 28] and others being almost static [29].

If holocentric clades have higher rates of chromosome number evolution, we might expect holocentric species to exhibit higher chromosome number. Anecdotal evidence does seem to suggest that some of the highest chromosome numbers observed are in clades with holocentric chromosomes. For instance, in insects, the highest chromosome numbers are observed in the holocentric order, Lepidoptera [6]. However, initial analyses have found no significant difference in chromosome number among holocentric and monocentric clades of insects [6]. This previous study was limited to an order level analysis and only tested whether the mean chromosome number among monocentric and holocentric clades was different. A stronger test of the impact of holocentricity would be to investigate the rates of fusions, fissions, and polyploidy in clades with holocentric and monocentric chromosomes.

**Fig 1:**
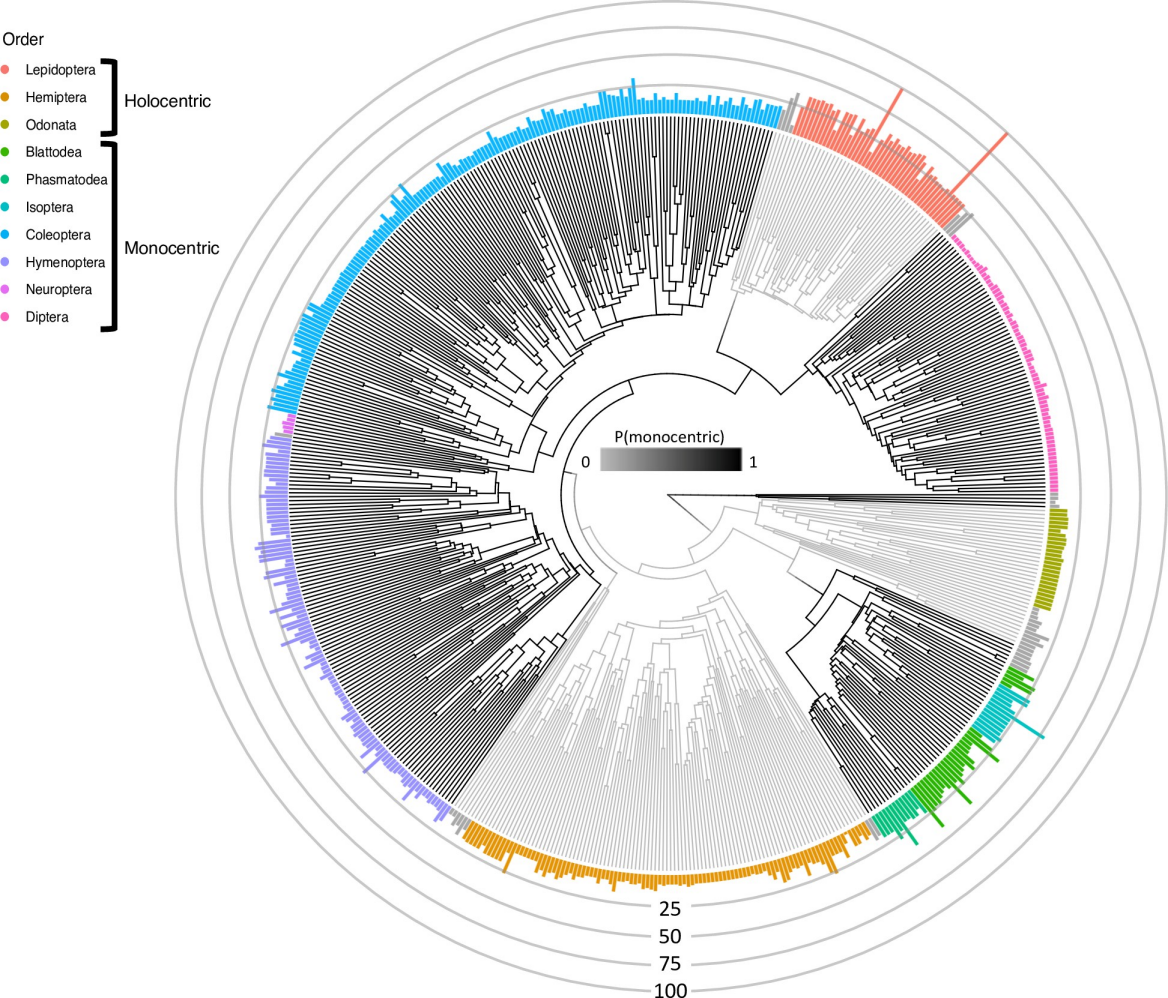
Phylogeny with type of centromere and chromosome number. The black branches represent orders with monocentric chromosomes and the gray branches represent orders with holocentric chromosomes. The height of the bars at the tips of the phylogeny represent the haploid chromosome number. The bar colors represent different insect orders and all grey bars are orders with fewer than 20 genera.

In this study, we used chromosome number and centromere type for 4,393 species belonging to 599 insect genera to test whether clades with holocentric chromosomes have a higher rate of chromosome number evolution than clades with monocentric chromosomes (Fig 1). We chose to use insects because they have multiple clades with monocentric and holocentric chromosomes, are incredibly speciose, and exhibit striking diversity in chromosome number [6, 30–33]. We hypothesized that clades with holocentric chromosomes should exhibit higher rates of fusions and fissions since these mutations should be less costly in these clades. However, we found no evidence for higher rates of chromosome number evolution in holocentric clades in comparison to monocentric clades. Instead, we found that Lepidoptera, a holocentric clade, exhibits some of the highest rates of chromosome number evolution, while other holocentric clades exhibit some of the lowest rates. Our results suggest characteristics other than holocentricity and monocentricity are key in determining rates of chromosome number evolution.

## Methods

### Data collection

We downloaded all available chromosome data for insects from a prior study [6]. This dataset is composed of 12,411 species comprising 376 families and 3,872 genera. The minimum haploid chromosome number is 2 while the maximum haploid chromosome number is 141. There are 3,465 species with holocentric chromosomes and 8,946 species with monocentric chromosomes. This paper also included classification of each order into either monocentric or holocentric. From this dataset, we extracted the homogametic haploid chromosome number for each of the species. We used genus level phylogenies from a previous study that contained 1,726 tips [34]. These trees were built using one of two backbones trees from previous studies [35, 36]. We downloaded two posterior distributions, each containing 100 trees, based on these backbone trees. These trees were used for all downstream comparative analyses. Our trait data set had an overlap of 599 genera with the phylogenetic data (Fig 1). In cases where we had multiple chromosome number records for genus, we retained all values and sampled from them as described below.

### Comparative analyses

We fit a model of chromosome number evolution on each tree from the posterior distribution (based on the Misof backbone). This model contains three mechanisms for changes in chromosome number: rate of chromosome number increase (fissions γ), rate of chromosome number decrease (fusions δ), and rate of whole genome duplication (polyploidy ρ). Each of these is estimated separately for clades with holocentric and monocentric chromosomes leading to six chromosomal rate parameters (S1 Fig). The final two parameters describe the transition to and from monocentric and holocentric (q_MH_ and q_HM_). We also fit a constrained version of this model that set the rate of polyploidy to zero. These models were specified using the R package chromePlus [5] and were fit using a Bayesian approach in the R package diversitree [37]. All analyses were completed in R version 3.6.3 [38] and scripts for all analyses are available in a GitHub repository (https://github.com/coleoguy/holocentric).

For each of the 100 trees in a posterior distribution, we randomly sampled tip states for genera with more than one record in our chromosome data set. By sampling across the chromosome dataset and the posterior distribution of trees we are able to account for both phylogenetic and tip state uncertainty. For purposes of model fitting trees were rescaled to unit length; however, all rates reported in the paper have been back transformed to be in units of millions of years. As is customary for Markov models like the one fit here the rates reported are lambda parameters that describe the expected waiting times for a transition to occur. Each Markov Chain Monte Carlo (MCMC) was initialized with parameter values drawn from a uniform distribution from 0 to 1. Preliminary analyses were conducted with a uniform prior and while most MCMC chains reached convergence quickly, a small fraction of runs would begin to sample very high rates that are biologically unrealistic. Given sufficient time we might expect these runs to eventually converge on the posterior distribution but this length of time can be large due to the relative flatness of the likelihood surface when rates are unrealistically high. To fix this problem, we applied an exponential prior with a shape parameter of 0.5. This is a relatively uninformative prior but does favor lower rates avoiding the problem described above. With application of this prior we found that the vast majority of MCMC chains reach convergence in less than 10 generations. We repeated the MCMC on all 100 trees at 50 generations each. We removed the first twenty-five generations as our burnin for each MCMC run and combined the postburnin portion of all MCMCs to create our estimate of the posterior distribution of model parameters. Because our central question is whether holocentric clades have higher rates than monocentric clades, we report our results in terms of a mean rate difference statistic, Δ*R_x_* where the subscript *x* indicates the rate parameter. For example, for the rate fissions (γ), for each post-burnin sample we calculated Δ*R_γ_* as

$$\Delta R_{\gamma }=\gamma_{holo}-\gamma_{mono}$$

In addition to estimating the magnitude of this statistic, we also reduced it to a simple test of the motivating hypothesis by comparing the 95% credible interval of Δ*R_x_* (i.e. the 95% highest posterior density) with zero. If the entire 95% credible interval of Δ*R_x_* is positive, we interpret it as support for a higher rate of chromosome number evolution in holocentric clades. If the entire 95% credible interval of Δ*R_x_* is negative, we interpret it as support for a higher rate of chromosome number evolution in monocentric clades. Otherwise, we conclude there is not support for a significant rate difference in monocentric and holocentric clades. This approach also allows us to compare across all trees in the posterior distribution even if some trees exhibit on average higher or lower rates.

We repeated similar MCMC analyses as above for the analysis of orders, because each order is fixed for either holo- or monocentricity. We only estimate one set of rate parameters in each clade, and only analyzed clades with more than 20 species matching genera in the phylogeny. The cutoff of 20 was chosen based on previous work that showed that with smaller datasets the ability to reliably infer rates decreases [5]. This led to 10 order level analyses; three of the included orders (Hemiptera, Lepidoptera, and Odonata) have holocentric chromosomes, and seven that have monocentric chromosomes. To compare rates among orders we compared the credible interval for each parameter among orders. Finally, we completed a bootstrap analysis to assess the impact of uncertainty in phylogeny and sampled tip states (S3 Fig and S1 Text).

## Results

### Alternative phylogenies

The phylogenies used for this study were built using two different backbone trees. The primary difference between these two trees is in the estimate of branch lengths. The Misof backbone favors more recent branching events than does the Rainford backbone. The total branch length of trees using the Rainford backbone are approximately 25% greater than those using the Misof backbone. To determine if this variation impacted our results, we fit our full eight parameter model to both sets of phylogenies. As expected, we inferred slightly different rates depending on which posterior distribution we used. Rates were on average lower when using the posterior sample based on the Rainford backbone [36] than when using the posterior sample based on the Misof backbone [35]. To investigate the impact this has on our inference we calculated the Δ*R* statistic for the rate of fissions, fusions, and polyploidy comparing holocentric and monocentric species. We found that the Δ*R* statistics had nearly identical distributions (S2 Fig). Based on this finding for the remainder of the paper we present results based on our analysis of the Misof tree.

### Monocentric and holocentric rates

**Fig 2:**
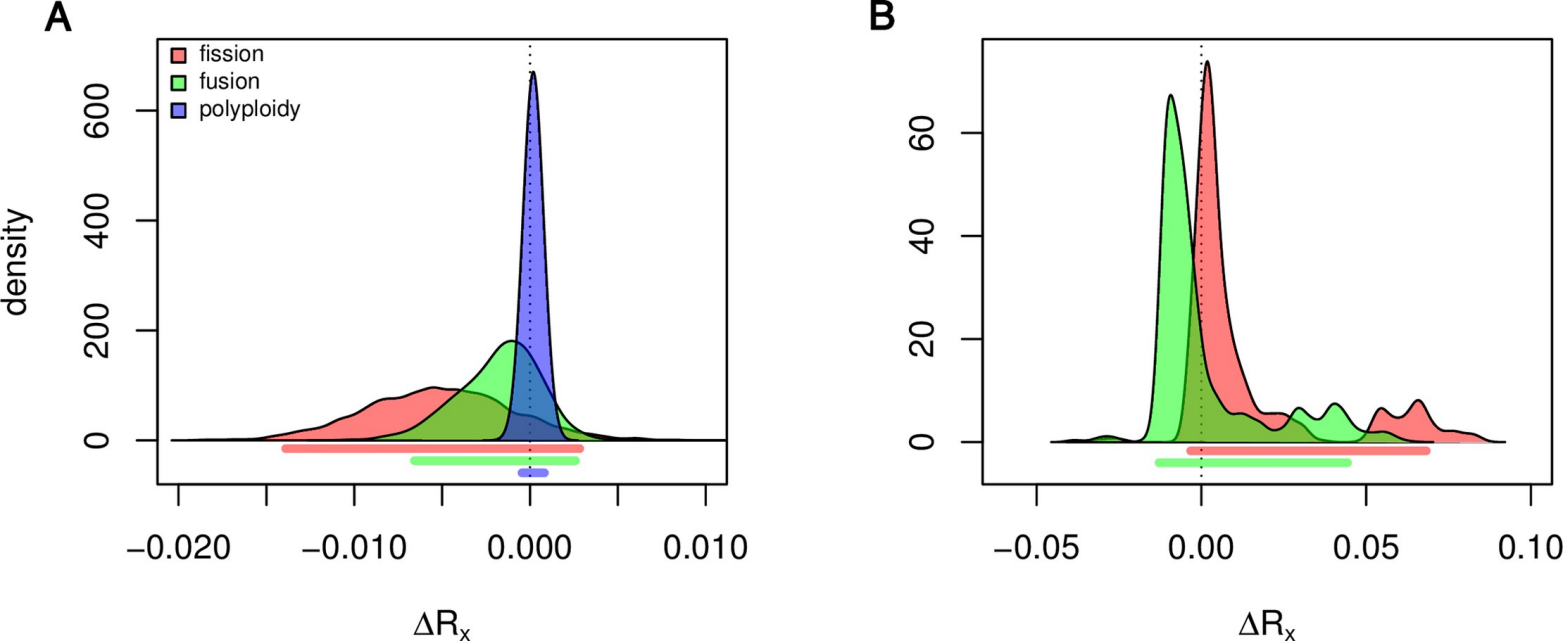
Rates of chromosome number evolution. Each curve represents the posterior distribution of the Δ*R_x_* statistic, where *x* is either fission, fusion, or polyploidy which is indicated by the color of the fill. Positive values of this statistic indicate that holocentric clades evolve more quickly than monocentric clades while negative values indicate that monocentric clades evolve more quickly than holocentric clades. Below the curves the lines indicate the 95% credible interval of each statistic. A) Results from fitting a model with all three possible transitions (fissions, fusions, and polyploidy). B) Results from fitting a model where chromosome number can change only through fissions and fusions. Under both models the credible interval of each parameter spans zero indicating no significant difference in rates of chromosome number evolution in clades with holocentric and monocentric chromosomes.

We explored two models for the evolution of chromosome number. The first model included fusion, fission, polyploidy, each estimated in holocentric and monocentric lineages as well as transitions between monocentric and holocentric chromosomes. The Δ*R_x_* statistics for fusions, fissions and polyploidy had credible intervals that overlapped zero (Fig 2A). This suggests that contrary to our hypothesis holocentric lineages do not have higher rates of chromosome number evolution. Because polyploidy events are likely rare, we also explored the impact of excluding polyploidy from the model. In this analysis we found qualitatively similar results. The credible interval of the Δ*R_x_* statistics again overlapped zero (Fig 2B). To assess the impact of uncertainty in phylogeny and sampling of possible chromosome numbers for each genus we performed a bootstrap analysis. We found that all bootstrap datasets were consistent with our empirical results (S3 Fig and S1 Text) suggesting that both sources of uncertainty (phylogeny and chromosome number) have little impact on rate estimates.

### Orders rates

**Fig 3:**
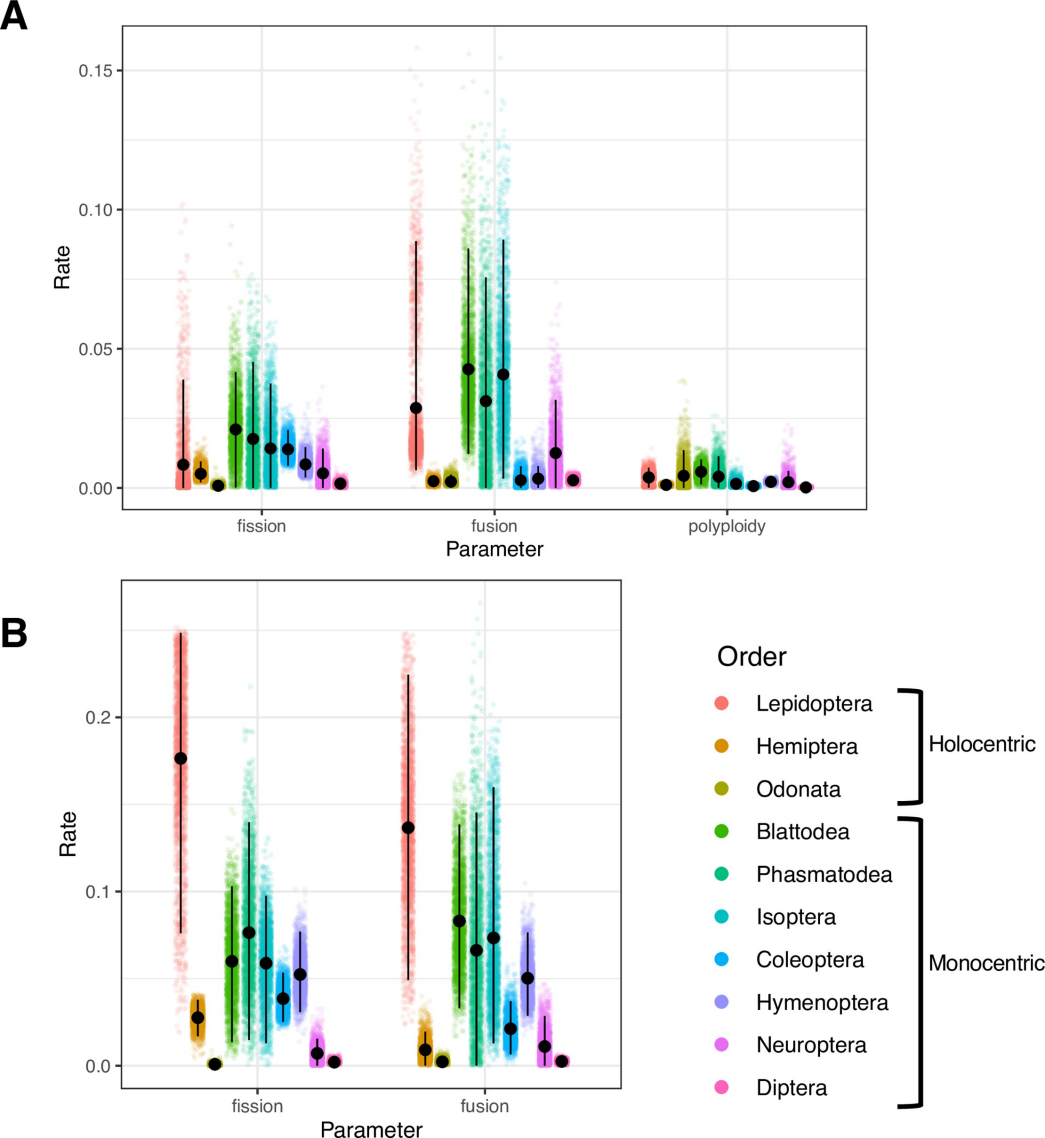
Rate of chromosome number evolution based on order. Rate estimates are plotted on the vertical axis and mechanisms on the horizontal. Each point is a single sample from the posterior distribution. Vertical black lines indicate the credible interval for each parameter. A) Full model results with fissions, fusions, and polyploidy. B) Constrained model with only fissions and fusions.

Rates of chromosome number were also estimated independently for each of the 10 orders with at least 20 genera in our phylogenetic dataset. Three orders (Hemiptera, Lepidoptera, Odonata) have holocentric chromosomes, while the other seven (Blattodea, Coleoptera, Diptera, Hymenoptera, Isoptera, Neuroptera, and Phasmatodea) have monocentric chromosomes. For this analysis we fit a complex model with fusion, fission, and polyploidy, and a simple model that excluded polyploidy. This order level analysis revealed striking differences in rates of fusion, fission, and polyploidy among orders, and distinct differences in rate parameters estimated under the two models. Under the complex model monocentric orders exhibited the highest rates of fissions, fusions, and polyploidy (Fig 3A). Under the simplified model Lepidoptera (a holocentric lineage) exhibited the highest rates of chromosome number evolution (both fusions and fissions) (Fig 3B). However, most monocentric orders exhibited intermediate rates and the other two holocentric orders exhibited some of the lowest rates of fusions and fissions. Taken together these results suggest that factors other than centromere type must be key in determining rates of chromosome number evolution in insects.

## Discussion

Lepidoptera have long been recognized as exhibiting striking variation in chromosome number [39]. The extreme distribution of chromosome number observed in lepidoptera has been a driving force in the development of the hypothesis that holocentricity allows for rapid changes in chromosome number [19, 20]. Our results find little support for this hypothesis. Looking across insects we find that rate estimates for holocentric and monocentric lineages are nearly equal. Our choice of models (either including or excluding polyploidy) impacted our rate estimates. When we fit the full model, we found that rates tended to be higher in monocentric lineages, but the reverse was true when we fit a model where polyploidy was not allowed. We note that though the credible interval of all Δ*R_x_* statistics overlapped zero in this simplified model 83% of the posterior distribution of Δ*R_γ_* (difference in fission rates) is above zero. We suggest that this may be a signal for a weak impact of holocentricity on rates of fission. However, when we investigated rates of chromosome number evolution within orders, we found that clades with holocentric chromosomes exhibited both some of the highest and lowest rates observed in insects. We propose that the variation in chromosome number within Lepidoptera is likely better explained by other traits that can impact the rate of chromosome number evolution (e.g. meiotic drive, polyploidy, phylogenetic history, and population sizes).

Meiotic drive is one possible driver of changes in chromosome number. We have recently shown that meiotic drive in mammals likely explains variation in rates of chromosome number evolution and the distribution of chromosome morphologies [5]. Work in mice has shown that meiotic drive is based on the strength of centromeres, where strength is characterized by the ability to express kinetochore proteins and interact with spindle fibers [40]. In this system, a fusion with the same centromere strength was shown to be either favored or disfavored depending on the genetic background that it was segregating within. In holocentric chromosomes, since they have a diffuse centromere, meiotic drive is thought to be less likely since multiple sequences must be favored simultaneously to have a strong impact on segregation [41]. Therefore, meiotic drive could potentially increase or decrease rates of chromosome number evolution. This may suggest monocentric clades exhibit more extreme rate variation dependent on the presence or absence of meiotic drive. This expectation matches well with the variation in rates that we observe under our complex model, where monocentric orders exhibit more variation in mean rates than holocentric orders (Fig 3A). However, our inference of rates under a simplified model show the opposite pattern with more variation in rates in holocentric orders (Fig 3B).

While fissions and fusions can make small changes to chromosome number, polyploidy events have the potential to lead to large increases in chromosome number much more rapidly. The frequency and impact of polyploid in insect genome evolution is still widely debated. Some analyses, for instance those based on distribution of ages among paralogs, suggest many whole or at least large-scale duplication events in at least 18 orders [42, 43]. In contrast, analyses based on synteny suggest fewer whole genome duplication events [44, 45]. Even a small number of polyploidy events depending on their distribution in the tree could lead to much higher variance in chromosome number for a clade. The application of probabilistic models that include polyploidy as a parameter are particularly important if the goal is to understand whether or not fissions and fusions are occurring at different rates among clades [5, 46–48]. The striking differences that we see in rate estimates under our two models is a clear example of the importance of evaluating the impact of polyploidy. However, we note that in our analysis the credible interval of our test statistic overlapped zero using both approaches. This suggests that the inclusion or exclusion of polyploidy in this particular analysis has no impact on our interpretation of the results (Fig 2).

Likewise, analyses within any one clade are difficult to interpret; for instance, the Reduviidae are a group of holocentric hemipterans. If holocentricity allows for tolerance of fissions we would predict that this clade would show large variations in chromosome number, but surveys of this group show that they have very little variation in chromosome number [49]. However, without a closely related clade with monocentric chromosomes, it is difficult to weigh the evidence against the traditional hypothesis for increased rates in holocentric clades. Furthermore, comparisons among studies is also difficult because rates are directly affected by divergence time estimates and the method of parameter estimation (e.g. the application of priors in Bayesian analyses). We argue that comparisons of rates are only informative in cases where a single phylogeny with a consistent approach to dating and rate estimation has been applied to both clades with holocentric and monocentric chromosomes.

One potentially important cause of variation in rates of chromosome number evolution is population size. This idea has its origins in the development of models of chromosomal speciation [2]. White proposed that most chromosomal rearrangements were underdominant and would be more likely to fix in small demes due to drift, and that these changes could then act as reproductive barriers when demes expanded their range and came into secondary contact [50]. This model of speciation likely is not representative of most diversity and has been shown to be unlikely under a range of potential parameter values [51]. However, White’s ideas led to an intense focus on predictors of chromosomal variation [1, 7, 52–56]. Many of these studies suggest that species or clades with small population sizes have higher rates of chromosome number evolution. Unfortunately, these were all completed prior to the robust development of comparative methods that can be applied to the evolution of chromosome number across large clades and some compared highly divergent clades. Explicitly modeling the impact of population size on estimated rates of chromosome number evolution within clades would be a significant advancement to our understanding of the determinant of rates of evolution.

Variation in chromosome number is highly heterogeneous across clades–some large clades are nearly static while other closely related clades show striking variation. This observation has been difficult to explain despite a century of investigation. We believe that the approach that we have used here modeling chromosome number and a possible explanatory variable simultaneously offer a way forward to finally determine what causes variation in rates of chromosome number evolution.

## Supporting information

S1 Fig Model for the evolution of chromosome number in monocentric and holocentric lineages. At an instance in time a lineage will have *i* chromosomes and either monocentric or holocentric chromosomes. A lineage can make four possible transitions: *δ* the fusion of two chromosomes, *γ* the fission of a chromosome, *ρ* a whole genome duplication, and a transition in centromere type (i.e. transition from monocentric to holocentric q_MH_ or transition from holocentric to monocentric q_HM_).
(TIF)

S2 Fig Comparison of inferences under alternative backbones. In each plot we show the Δ*R* statistic for the three parameters of interest in our model. We find that regardless of the backbone phylogeny the resulting statistic has a largely similar distribution. Black lines represent the statistic estimate using the Misof backbone while red lines represent the statistic estimate using the Rainford backbone.
(TIF)

S3 Fig Comparison of bootstrap and empirical estimates. In each plot we show the Δ*R* statistic for one of the parameters of interest in our model A) fissions, B) fusions, and C) polyploidy. In each plot colored lines show the density distribution of 1000 bootstrap datasets. The black dashed lines show the density distribution from the empirical dataset. The solid black line at the bottom of each plot shows the limits of the most extreme credible intervals from all 1000 bootstraps. If a bootstrap dataset conflicted with our empirical analysis it would have a credible interval where the lower value was greater than zero or its higher value was less than zero. All 1000 credible intervals span zero.
(TIF)

S1 Text Impact of uncertainty in phylogeny and chromosome number. Discussion of bootstrapping to assess uncertainty in results due to phylogeny and tip states.
(PDF)

S1 Table Sample sizes and parameter estimates. In the first column we list groupings for which we estimated rates. No rate estimates are given for the final 12 orders because the sample size fell below our threshold for inclusion in the order-based analysis.
(PDF)
