# Supplementary figures and images for: Chromosome number evolves at equal rates in holocentric and monocentric clades
Source: PLoS Genet. 2020 Oct 13;16(10):e1009076. doi: 10.1371/journal.pgen.1009076 (PMC7584213; doi:10.1371/journal.pgen.1009076)

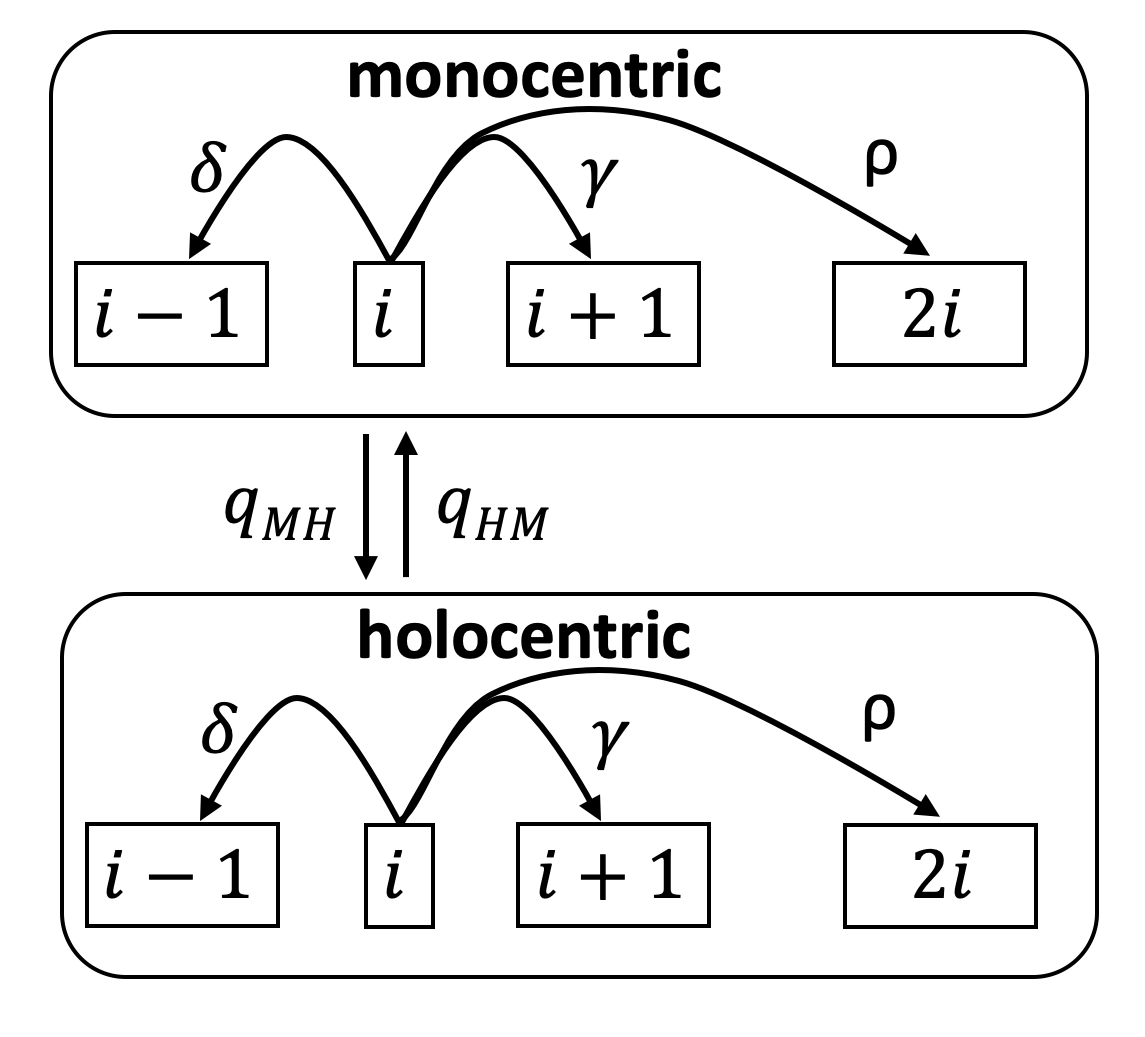

Supplement: S1 Fig — At an instance in time a lineage will have i chromosomes and either monocentric or holocentric chromosomes. A lineage can make four possible transitions: δ the fusion of two chromosomes, γ the fission of a chromosome, ρ a whole genome duplication, and a transition in centromere type (i.e. transition from monocentric to holocentric qMH or transition from holocentric to monocentric qHM). (TIF) [file pgen.1009076.s001.tif]

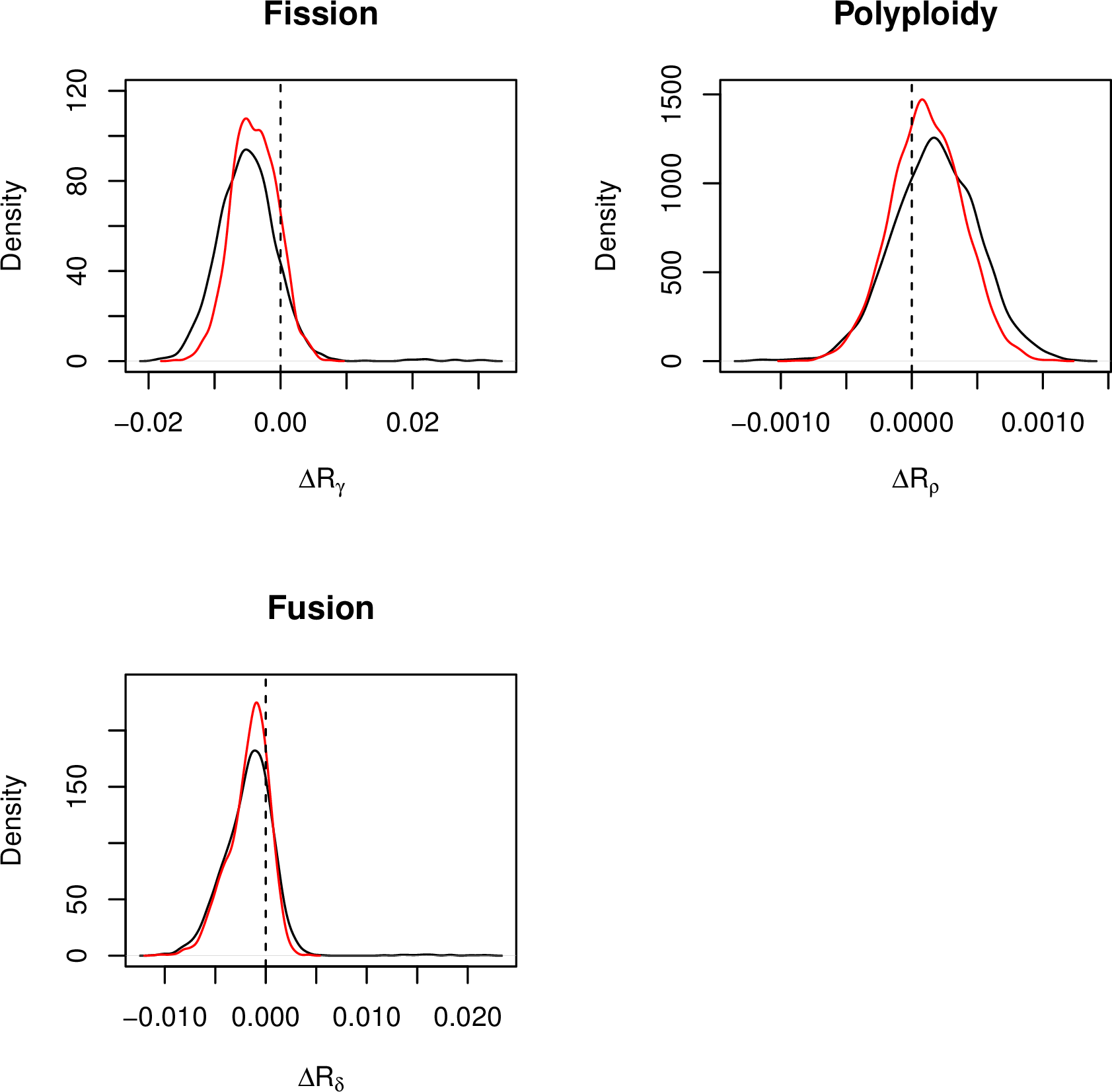

Supplement: S2 Fig — In each plot we show the ΔR statistic for the three parameters of interest in our model. We find that regardless of the backbone phylogeny the resulting statistic has a largely similar distribution. Black lines represent the statistic estimate using the Misof backbone while red lines represent the statistic estimate using the Rainford backbone. (TIF) [file pgen.1009076.s002.tif]

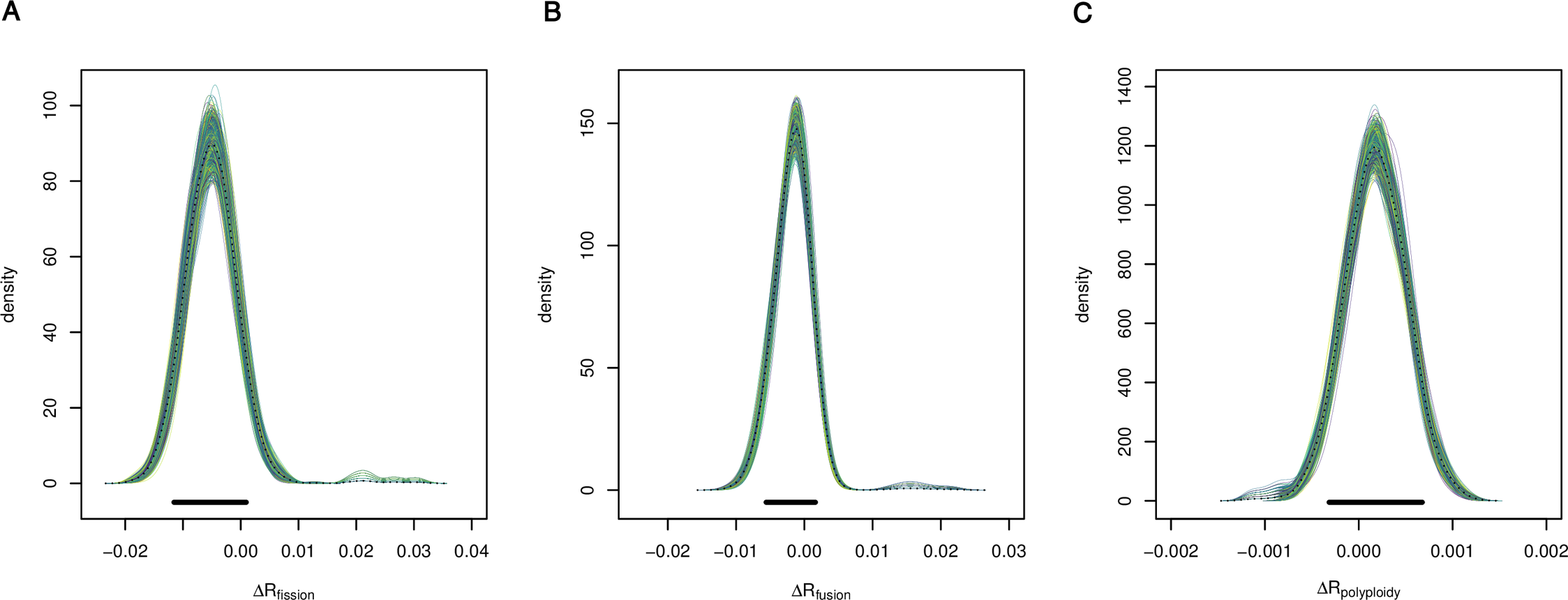

Supplement: S3 Fig — In each plot we show the ΔR statistic for one of the parameters of interest in our model A) fissions, B) fusions, and C) polyploidy. In each plot colored lines show the density distribution of 1000 bootstrap datasets. The black dashed lines show the density distribution from the empirical dataset. The solid black line at the bottom of each plot shows the limits of the most extreme credible intervals from all 1000 bootstraps. If a bootstrap dataset conflicted with our empirical analysis it would have a credible interval where the lower value was greater than zero or its higher value was less than zero. All 1000 credible intervals span zero. (TIF) [file pgen.1009076.s003.tif]
