# Supplementary material for: Chromosome number evolves at equal rates in holocentric and monocentric clades
Source: PLoS Genet. 2020 Oct 13;16(10):e1009076. doi: 10.1371/journal.pgen.1009076 (PMC7584213; doi:10.1371/journal.pgen.1009076)
Supplement: S1 Text — Discussion of bootstrapping to assess uncertainty in results due to phylogeny and tip states. (PDF) [file pgen.1009076.s004.pdf]

One potential concern with our analysis approach is that we are using a phylogeny with only a single tip for each genus included in our analysis, but in many cases, we have multiple species in a given genus and sometimes they vary in chromosome number. Our solution was to randomly sample from all species in a given genus and assign one of the observed chromosome numbers for each genus tip in our phylogeny. Using these sampled chromosome numbers, we then estimated rates for the current tree. Next we repeated this process for each of the 100 trees from the posterior distribution. Finally, we combined the post-burnin portion of our MCMC performed on each tree to generate a posterior distribution incorporating uncertainty in both phylogeny and tip state. This combination of a sampled tree from the posterior and a sample of possible chromosome number assignments to each genus will be referred to as a sample set below. To assess the impact uncertainty in trees and chromosome number data we conducted a bootstrap analysis with 1000 replicates. Briefly, for each bootstrap replicate we took our existing MCMC log files and chose 100 of them with replacement. This led to an average of 63 sample sets being used for parameter inference and in most (greater than 90%) bootstrap replicates one or more sample set was included four or more times. With this approach if some sample sets lead to very different answers we expect to see variation in our calculation of the delta R statistic that is reported in the paper. Our results (S3 Fig) indicate that sample sets have no significant impact our inference approach. None of the 1000 bootstrap replicates led to inferences different from those reported in the main body of the manuscript. More broadly we would suggest that this type of bootstrap replicate should become a more
