# Supplementary material for: Chromosome number evolves at equal rates in holocentric and monocentric clades
Source: PLoS Genet. 2020 Oct 13;16(10):e1009076. doi: 10.1371/journal.pgen.1009076 (PMC7584213; doi:10.1371/journal.pgen.1009076)
Supplement: S1 Table — In the first column we list groupings for which we estimated rates. No rate estimates are given for the final 12 orders because the sample size fell below our threshold for inclusion in the order-based analysis. (PDF) [file pgen.1009076.s005.pdf]

Supplemental Table 1. Sample sizes and parameter estimates. In the first column we list groupings for which we estimated rates. No rate estimates are given for the final 12 orders because the sample size fell below our threshold for inclusion in the order-based analysis.

**Groupings**	**[1]Chrom. Records**	**[2]Records genera on tree**	**[3]Genera on tree**	**Model with Polyploidy mean (95% credible interval)**			**Model w/o Polyploidy mean (95% credible interval)**	
				Fusion	Fission	Polyploidy	Fusion	Fission
**Holocentric**	**3465**	**1000**	**195**	**0.0034 (0.0010-0.0055)**	**0.0099 (0.0045-0.0163)**	**0.0012 (0.0008-0.0016)**	**0.0212 (0.0106-0.0437)**	**0.0416 (0.0293-0.0691)**
**Monocentric**	**8946**	**3393**	**404**	**0.0050 (0.0007-0.0095)**	**0.0149 (0.0101-0.0200)**	**0.0010 (0.0006-0.0014)**	**0.0196 (0.0006-0.0282)**	**0.0278 (0.0005-0.0367)**
Blattodea	*111*	*66*	*27*	0.0426 (0.0123-0.086)	0.021 (0.0002-0.0416)	0.0058 (0.0013-0.0102)	0.0831 (0.0329-0.1384)	0.0599 (0.0135-0.103)
Coleoptera	*4393*	*1424*	*157*	0.0027 (0.0000-0.0078)	0.0139 (0.008-0.0208)	0.0006 (0.0001-0.0013)	0.0212 (0.0065-0.0371)	0.0385 (0.0252-0.0534)
Diptera	*2106*	*1138*	*70*	0.0028 (0.0012-0.0046)	0.0016 (0.0000-0.0028)	0.0002 (0.0000-0.0005)	0.0025 (0.0008-0.0044)	0.002 (0.0006-0.0039)
Hemiptera	*1695*	*470*	*103*	0.0024 (0.0005-0.0042)	0.0051 (0.002-0.0095)	0.0011 (0.0005-0.0017)	0.0091 (0.0001-0.0196)	0.0276 (0.0169-0.0379)
Hymenoptera	*1598*	*622*	*106*	0.0033 (0.0000-0.0079)	0.0085 (0.0038-0.0146)	0.0022 (0.0012-0.0033)	0.0502 (0.0287-0.0765)	0.0524 (0.0307-0.077)
Isoptera	*81*	*46*	*13*	0.0408 (0.0033-0.0892)	0.0142 (0.0000-0.0375)	0.0015 (0.0000-0.0036)	0.0734 (0.0128-0.1598)	0.0588 (0.0129-0.0975)
Lepidoptera	*1132*	*322*	*45*	0.0287 (0.0064-0.0887)	0.0083 (0.0000-0.0389)	0.0038 (0.0001-0.0073)	0.1366 (0.0492-0.2245)	0.1764 (0.0761-0.2484)
Neuroptera	*89*	*33*	*5*	0.0125 (0.0000-0.0316)	0.0053 (0.0000-0.0142)	0.0021 (0.0000-0.0061)	0.011 (0-0.0285)	0.0071 (0.0000-0.0155)
Odonata	*413*	*157*	*25*	0.0022 (0.0005-0.0048)	0.0008 (0.0000-0.0018)	0.0044 (0.0000-0.0135)	0.0022 (0.0004-0.0048)	0.0008 (0.0000-0.0019)
Phasmatodea	*89*	*24*	*12*	0.0312 (0.0000-0.0757)	0.0176 (0.0000-0.0453)	0.004 (0.0001-0.0114)	0.0662 (0.0000-0.1454)	0.0764 (0.0147-0.1398)
Collembola	*76*	*7*	*4*					
Dermaptera	*49*	*11*	*3*					
Ephemeroptera	*7*	*7*	*6*					
Mantodea	*101*	*10*	*3*					
Mecoptera	*15*	*12*	*3*					
Orthoptera	*276*	*8*	*3*					
Phthiraptera	*16*	*4*	*2*					
Psocoptera	*94*	*10*	*4*					
Raphidioptera	*5*	*3*	*1*					
Thysanoptera	*24*	*4*	*2*					
Tricoptera	*34*	*14*	*4*					
Zoraptera	*1*	*1*	*1*					

[1] "Chrom. records" is the number of species in a given group for which chromosome number was available. [2] "Records genera on tree" is the number of species with chromosome data that match a genus on the phylogeny used for our comparative analysis. [3] "Genera on tree" is the number of genera tips in each grouping on our phylogeny.
